# Suppression of Consumption

**Published:** 1902-01

**Authors:** R. C. Bankston

**Affiliations:** Birmingham, Ala.


ATLANTA

### Journal-Record of Medicine.

Successor to Atlanta Medical and Surgical Journal, Established 1855, and Southern Medical Record, Established 1870.

Vol. III. JANUARY, 1902. No. 10.

BERNARD WOLFF, M.D., EDITOR, Nos. 319–20 Prudential.

Published Monthly.

M. B. HUTCHINS, M.D., BUSINESS MANAGER, No. 64 Marietta St.

### ORIGINAL COMMUNICATIONS.

### SUPPRESSION OF CONSUMPTION.

By R. C. BANKSTON, M.D.,
Birmingham, Ala.

In addressing you on suppression of consumption I desire to consider it in a practical way which will put the laity in appreciative touch with our ideas, keeping studious attention upon this important medical subject. Nothing is more necessary to the welfare of man than knowledge of the disease in various forms. Will we successfully cure it? I answer yes, and the best route to that end is through agitation and not give precedence to unimportant issues involving no principle of consequence. Though the subject may be worn from time serving, and the ground covered herein, old and familiar, yet I desire to reiterate: the object sought to be impressed cannot be talked too much. Any suggestion, however vague, calling attention and serving to keep the fire of interest in its study alight is commendable, that scientific investigation may be pursued and certain cure accomplished. No one is prepared to speak authoritatively, but the question is so vital that we should from environment feel peculiarly interested. Much has been written upon methods of cure. After close study of the devitalized state of those susceptible to onslaught, we come to this conclusion: Vigorous, well nourished tissue is not favorable to the reception and manifestation of the germ, which accepted medical opinion designates the cause of the disease. A serious thought enters for consideration here: May not the cause be a degenerative process or physiology below par and the germ an incident of the condition? Of course they are invariably found in all manifestation, but the multitude who are largely exposed without contracting it suggests the possibility of present theory being reversed in application. This is not an opinion asserted, but argument; however, I do assert that it is preventable, and under proper conditions is curable, and will yet be exterminated. Continued agitation arouses study which, as education will demonstrate, preventive. While spread mainly by the poor, the rich contract it also; therefore we may expect community action in its elimination. The rich naturally are the educated class, and realizing the danger will support proper measures for suppression. Vital interest or self-preservation is too strong in life to presume upon any other course actuating. The greatest focus of the disease in Alabama is in Jefferson county. I allude to the Pratt Mines Prisons, where all are thrown together under circumstances which lower vital resistance, rendering them each prey to the virile implacable foe which does not let up on its victim usually, but conquers to a finish; however, it does not always finish such victim before the expiration of his prison term. In fact it is a practice when the disease has become apparent and days of labor are over; a sympathetic appeal for clemency is proposed and the pardoned sufferer unwisely turned loose on an unsuspecting public to scatter broadcast these virile germs to infect all who are susceptible, or is returned to the walls of the penitentiary, where he may infect any who may be receptive there. If the poor fellow was a criminal menace before incarceration, from vicious proclivities, how much greater as a danger now when he is capable of infecting with this horrible scourge all who are devitalized, who may come within the sphere of virility of this potency of destruction. If the people of Jefferson county do not take some steps to have this hot-bed of contagion abolished, the consequence will eventually be more serious through general infection than would freeing the entire horde of depraved non-infected criminals, all of whom, if they remain long enough, may contract and distribute it. Human knowledge has never realized a more unconquerable disease, but with present information, we are nearing the time when this foe will be vanished and mankind freed from fear and thraldom more iniquitous than any ever known. This subject has left the field of the intangible and is exciting a degree of interest unequalled by any other proposition before the world. Means advanced are meeting with success sufficient to excite hope that proper precautions are being taken and an infallible antidote will be found to successfully eradicate consumption from the list of life's fears; whether it be through elimination of germs or increasing vital resistance is not relevant, however; the way to achieve it is through continuous study. Realization through education broadens mind, quickens knowledge and gives the faculty of comprehension needed to appreciate cause productive and methods required to eliminate. The theory of contagion is old, but is only now impressing itself as important and correct. It is one of the diseases affecting man and animal alike. While contagion has been admitted for ages, it was only about thirty years ago that the germ was isolated and the important discovery made that it is due to bacilli, which are introduced into the system through the alimentary canal, the respiratory tract or the skin, provided conditions are favorable and pabulum present for its peculiar requirement. A person in good health cannot be impressed under ordinary circumstances, but by keeping germs ever present any one may become devitalized from some cause, at which time he may be impressed; therefore it stands to reason that any whose duties call them into the localized zone of germ activity would better feel sure that vital powers are above par, and not in condition for germ propagation to be effective. I know it is preventable, all necessary being to avoid the focus when devitalized. Present conclusions warrant views which find expression here, and we do not believe it will be many years before obstacles are surmounted and conditions of the disease understood. The detriment of the present is ignorance of laws of health and pathology of disease. Overcome them and a great hindrance to stamping it out will have been removed. Ten years ago a commission of British savants appointed to study it made the following as part of their report, which stands today as basis for general opinion on it, though it does not speak well for progressiveness. First, consumption is contagious. Second, the contagion is a living germ. Third, they can only grow and propagate in an animal body where fluids are favorable as pabulum. Fourth, the principal source of infection is the expectoration from the lungs of sufferers which, allowed to dry, becomes pulverized and, as dust, will float in the air and be conveyed to the nostrils for inhalation, or will settle on milk, water or food and be carried into the body, or may contaminate an animal which may be killed and used as food before any evidence of infection becomes apparent. Milk likely is a common source of infection, being used in its natural state, while thoroughly cooking meat destroys the germs largely. This theory you may observe is not new. During the past five years its intelligent application has domonstrated its value. I believe it correct. All needed to determine it is to educate sentiment to knowing its merit. While these general causes must be considered in any plan devised to stamp it out, yet our greatest danger is in ignorant commingling with infected individuals from whom the disease may be directly spread to those receptive; from devitalised health. To explain at more length, no one, however robust, but may at some time in life have pneumonia or other disease or an occupation and environment which would temporarily devitalize blood and tissue. Suppose while in such state one should be brought in contact with the germ, conditions being right, the field is planted and a manifestation results. These statements only need study to be verified. For some years, but in an isolated way, doctors have been making suggestions which have not met with deserved recognition because the importance is not understood. People are not prepared to comprehend the significance of such propositions and realize that the future of man is largely enveloped in remote problems. Such being fact, these menaces should not longer exist, but removed and replaced by knowledge of cause and importance of arrest that health may be assured and the span of life lengthened. Take an infected person who has been educated sufficiently to know the danger existing with himsel, as a focus for infecting any who may be receptive, with whom he may come in contact; qualify him to protect by educating him briefly in the course he should adopt, and you will have done much to lessen its spread. A campaign of education will do more to stamp it out than all other policies advanced. Base your efforts on knowledge and the result will be tangible. No effective suppression can be employed until the masses are educated to comprehend the danger and know that blind chance is an unsafe guide· Its rapid increase makes it a vital problem. Ignorant people who know nothing of their danger serve as means for its continuance. When we realize that each individual, however conversant with the infection, may become run down and contract it through constant germ presence, that known, should arouse interest. It should be studied and laws passed which can be applied vigorously that all sources of infection may be stamped out and cases existing isolated.

Such course would be of immense value, with other laws making strict physical examination necessary before any, either citizen from abroad or foreign, can enter our country to endanger us. That course would involve both trouble and expense. We admit it, yet the money spent and time taken to stamp out a curse, responsible directly and through inherited devitality for nearly half the total death rate, would be well invested. Let an epidemic of cholera or yellow fever start our way and we are up in arms to repel. Millions will be spent in active quarantine with no regret, while great congratulation is indulged if successful, yet we have this insidious foe in our midst which is destroying more life than all epidemics of every other infectious disease combined and we do not hear a word of alarm, or protest, from the public. Why? Because they do not know their danger. Attention has not been attracted in a way to impress its alarming character. Why wait until the disease through heredity becomes so general that we will be a nation of consumptives. I have not the statistics at hand to verify it, yet the progressive increase of consumption compared with total population is alarming. It is not making an extreme assertion to say within fifty years, unless something is done to arrest it, more than half the death rate will be of this disease. If all were educated so that knowledge of it would be general, then the importance to abate would be appreciated and eradication accomplished. Those who know the danger should unite to fight it. If undertaken intelligently it will be stamped out and relegated to history. For some years various State and county boards of health have had this subject under consideration. Their work has been such that no result is perceptible. It is wanting in co-ordinative spirit. Our government should take hold of the proposition and pass laws restricting disease through heredity. I do not wish to be misunderstood. It cannot be transmitted a heritage *per se*, but the devitalized tissue favorable to its continuance can be, and one so constituted will sooner or later come into germ presence which finding favorable pabulum takes possession of the field and grows. This idea while largely theoretical is admitted reasonable. In fact, no theory disdaining reason can expect to develop by imaginative faculty while it holds all philosophy incomplete that bounds inquiry to the limit of the certain. Conjecture and induction contribute hypothetically to make the essence of philosophical conclusion. We know enough of the disease to advance our theory of its cause, and means which if adopted will certainly remove it. No legislation now advocated would accomplish good, because the principle underlying is not understood. What those interested should do is to get our government to appoint a commission of five physicians who should give the subject exhaustive study at various places, then meet, devise and perfect plans which could be suggested to the government for execution, which would be effective in result. The following suggestions worked out might give a basis for a feasible plan which would be successful. First, the United States Government to make a liberal appropriation to cover the expense of the board and give every facility and five years' time for them to arrive at and promulgate a finding. Second, each State to co-operate and appoint a board of five physicians, the State boards to compile their information and report to the government board after three years, and annually thereafter. Third, each county to organize medical societies which shall have charge of local health conditions and select three members as a committee to investigate all cases reported, see that they are properly isolated and kept under best physiological conditions appropriate to overcome the disease and its spread in the community. Each county shall appropriate means to cover the expense of its commission. Each State shall appropriate for its commission, who shall be responsible to the commission of the government, which shall call conferences with the various State commissions, to be represented by delegates selected for fitness and familiarity with the subject and its importance. This body to extend invitation to all who may feel interested to come before it and give their views; as the object is educational, all finding should be extensively published and the literature scattered over the country to attract attention and obtain the co-operation of all in the systematic study of the disease and measures devised to stamp it out. The government to make annual appropriation to cover the cost of the sittings. The commission or college of science to be under the supervision of a chief who shall be head of the department of public health and statistics, tenure of office should be ten years, and so arranged at the beginning that one member should go out every second year. They should give their whole time to the affairs of the position, and not be eligible to reappointment after having served ten years. New blood infused means vigor and development. The office of chief of the department of health to be a cabinet position, subject to prevailing rules and always filled by a physician. The commission or college of science should study public health and causes prevailing that would modify it. Their standing and *ensemble* should be par to that of the supreme court. Position on the commission should be a distinction sought as a rare honor and only awarded after the high attainment, learning and scientific ability of the aspirant should be known. The highest qualification should be imperative; otherwise the result will not be of character to complete the great work of eliminating degeneracy, eradicating disease and perfecting vital powers for continued health.

I hope this brief outline will serve suggestively as a synopsis of what should be undertaken; otherwise posterity will become so generally infected that extinction of entire nations is probable.

This subject is more vital in its relation to posterity than any other. We owe it a sacred duty to do something for relief. We should consider it a trust received from ancestry to study and devise means for eradicating this scourge, and not let it descend through the generations as a heritage of iniquity bequeathed as vicious taint by an ancestry conspicuous in history more for imperfection than for measures for the good of life. In any proposition hitherto advanced, few important factors enter. If we would achieve success, we should improve and strengthen vital resistance and educate all in the science of life. Let them know why we live—what we live for as a high purpose—how to live and get best result through familiarity with the laws of biology, physiology, hygiene, chemistry, philosophy and psychology. The first and last being the most important. I do not mean the psychology of the mountebank, but an understanding of the relations of mental, physical and reflex approximations and their harmony, giving a high quality and enduring principle of life. If all laws bearing on longevity were properly observed man would have no disease and would live out an expectancy based upon the amount of vital force with which he would be endowed at the beginning. Roll back obscuring shadows from the doctor's path, cast by indifference; bring forward the bright light of education and let it shine upon intelligent people, who, by cultivating intellect, have emancipated the world from the horrors of consumption, they contemplate the happy condition to exist perspectively compared with present distress, and let your earnest effort be to suppress consumption.

